# *Giardia duodenalis* multi-locus genotypes in dogs with different levels of synanthropism and clinical signs

**DOI:** 10.1186/s13071-020-04496-2

**Published:** 2020-12-02

**Authors:** Mathilde Uiterwijk, Lapo Mughini-Gras, Rolf Nijsse, Jaap A. Wagenaar, Harm W. Ploeger, Frans N. J. Kooyman

**Affiliations:** 1 Department of Infectious Diseases and Immunology, Faculty of Veterinary Medicine, Utrecht University, Utrecht, The Netherlands; 2 Present Address: National Reference Laboratory, The Netherlands Food and Consumer Product Safety Authority (NVWA), Centre for Monitoring of Vectors (CMV), Wageningen, The Netherlands; 3 Wageningen Bioveterinary Research, Lelystad, The Netherlands; 4 National Institute for Public Health and the Environment (RIVM), Centre for Infectious Disease Control (CIb), Bilthoven, The Netherlands; 5 Institute for Risk Assessment Sciences (IRAS), Faculty of Veterinary Medicine, Utrecht University, Utrecht, The Netherlands

**Keywords:** Giardiosis, Assemblage, Canine, Zoonotic, Cysts per gram

## Abstract

**Background:**

In dogs, infections with *Giardia duodenalis* are mainly caused by assemblages C and D, but also by the potentially zoonotic assemblages A and B. The aims of this study were to assess differences in assemblages (i) between dogs living mainly in close proximity to humans (synanthropic dogs) *versus* dogs living mainly among other dogs, (ii) between samples of dogs with or without loose stool, and (iii) related to the amount of cysts shedding.

**Methods:**

One hundred eighty-nine qPCR *Giardia* positive fecal samples of dogs originating from four groups (household, sheltered, hunting, and dogs for which a veterinarian sent a fecal sample to a diagnostic laboratory) were used for genotyping. For this, multi-locus genotyping of beta-giardin, triose phosphate isomerase, and glutamate dehydrogenase and genotyping of *SSU rDNA* gene fragments were performed. Fecal consistency was scored (loose or non-loose stool), and cysts per gram of feces were determined with qPCR.

**Results:**

Assemblage D was the most prevalent in all groups, followed by the other canid assemblage C. Also, mixed C/D was common. In two (synanthropic) household dogs, the potentially zoonotic assemblage AI was present. Although occurrence of assemblage AI in household dogs was not significantly different from dogs living among other dogs (sheltered and hunting dogs), it was significantly higher compared to dogs for which a sample was sent to a diagnostic laboratory. Dogs with assemblage D shed significantly more cysts than dogs with other assemblages (except for mixed C/D results) or dogs in which no assemblage could be determined. None of the assemblages was significantly associated with loose stool.

**Conclusion:**

Not only do dogs mainly shed the canid *Giardia duodenalis* assemblages D and/or C, the numbers of cysts per gram for the canid assemblage D were also higher than for the potential zoonotic assemblage AI. Based on the assemblages shed by dogs, the risk to public health posed by dogs is estimated to be low, even though the dogs that shed AI were synanthropic household dogs. Loose stool in infected dogs was not associated with any particular *Giardia* assemblage.

## Background

*Giardia duodenalis* (synonyms *G. intestinalis* and *G. lamblia*) is a common gastrointestinal parasite in humans and animals worldwide. This species can be further divided into eight assemblages denoted with letters A to H, based upon substantial sequence differences [1, 2].

Assemblages C to H are thought to have narrow host ranges, even though recent reports suggest that their host specificity might be broader than originally thought [3, 4].

Assemblages A and B are detected in humans in addition to several animal species, allowing for *G. duodenalis* to be considered a zoonotic agent [5]. Due to more recent insights into multi-locus genotypes (MLG), which further divide assemblages A and B in sub-assemblages with varying host ranges, the zoonotic importance of *G. duodenalis* is currently considered to be lower than previously thought [6–9].

Assemblages C and D are canid-specific. However, other assemblages, particularly A and B, can also be found in dogs [10–13]. The relative contribution of assemblage AI in synanthropic animals (defined as animals that live in close contact with humans), including dogs, can be as high as in humans themselves [6]. In contrast to this, other studies report predominantly canid assemblages in synanthropic dogs [8, 14, 15]. Use of multiple loci for detection of *G. duodenalis* has shown that mixed assemblage infections occur regularly in humans and many animals, including dogs [8, 16–19].

Although *Giardia* infections can cause gastrointestinal disease in dogs, there is no significant association of *Giardia*-positive fecal samples with diarrhea or loose stool [20–23]. However, the relation between fecal consistency and *Giardia* positivity has not been assessed as assemblage-specific. Also, the amount of cyst shedding has not been assessed assemblage-specifically, so it is not clear whether infections with canid assemblages C and D have a different host-pathogen interaction in dogs than infections with other assemblages, such as A and B. Results from an experimental study showed that dogs inoculated with trophozoites and cysts of human isolates developed no symptoms. However, dog numbers were low, and the isolates were not molecularly characterized [24].

Herewith, we aimed to determine whether synanthropic dogs are more likely to shed non-canid assemblages than dogs living among other dogs and whether infection with non-canid assemblages results in similar symptoms and numbers of cysts that are excreted. For this, *G. duodenalis*-positive dog fecal samples from four groups of dogs with different levels of synanthropism and differences in clinical signs were genotyped and the level of cyst shedding was determined.

## Methods

### Dogs and diagnostic tests

For this study, 189 *G. duodenalis* qPCR-positive dog samples obtained from previous studies were used. For more details about the study population, fecal sampling, DNA isolation, qPCR, rapid enzyme immunochromatographic assay (IDEXX SNAP^®^ *Giardia*), direct immunofluorescence (DFA, Merifluor) and centrifugation sedimentation flotation (CSF) coproscopical analysis, we refer to those studies [20, 25]. The household dogs (*n* = 47) were considered synanthropic dogs, whereas the sheltered (*n* = 48) and hunting dogs (scent hounds; *n* = 30) were considered group-housed dogs, living predominantly among other dogs. The dogs for which a sample was sent to the Veterinary Microbiological Diagnostic Center (VMDC) of the Faculty of Veterinary Medicine of Utrecht University (*n* = 64) were left out of the synanthropic group, because detailed information about living conditions was not available for all dogs. This group will be further referred to as ‘clinical dogs.’ Fecal samples of the clinical dogs were submitted for parasite testing to diagnose a possible parasitic cause of clinical symptoms (mostly gastrointestinal), but also, to a lesser extent, for control of antiparasitic therapy or routine monitoring. Fecal samples were scored for consistency and classified as loose or non-loose as described [20].

### Multilocus genotyping

MLG of *gdh* (glutamate dehydrogenase), *bg* (beta-giardin), and *tpi* (triose phosphate isomerase) and genotyping of *SSU rDNA* (small subunit ribosomal DNA) gene loci was performed on the fecal samples by nested PCRs. See Additional file 1: Table S1 for primer sequences, amplicon size, and references. The nested PCRs on single-copy loci were performed with DreamTaq DNA polymerase (Thermo Scientific) as described below, with several adjustments for optimization reasons. Nested PCR on *gdh* was performed as described [26] with the following modifications: bovine serum albumin (0.5 mg/ml) was included in the PCR mixture, and the annealing temperatures were 57.5 °C and 60.0 °C for the first and second amplification, respectively. Nested PCR on *bg* was performed as described [27] with an annealing temperature of 65.0 °C and 50.0 °C for the first and second amplifications, respectively. The first amplification of nested PCR on the *tpi* locus was performed as described [28], but with annealing temperature of 60.0 °C. The second amplification was assemblage-specific. Assemblage A-specific *tpi* amplification was achieved as described [19], but with an annealing temperature of 60.0 °C. Assemblage B-specific *tpi* amplification was achieved as described [29], but with an annealing temperature of 60.0 °C. The nested PCR on the *SSU*-*rDNA* locus was performed with Phusion Hot Start II DNA Polymerase (Thermo Scientific) with the buffer for GC-rich templates and the inclusion of 8% DMSO. The primers were those described [30], and the annealing temperature was 65.0 °C. In all PCRs, positive and negative control templates were included. Templates from both assemblage A and B were included in both assemblage-specific PCRs. All amplicons were Sanger-sequenced at BaseClear (Leiden, The Netherlands). The sequences were aligned and a phylogenetic tree was constructed in DNASTAR Lasergene 14, together with reference sequences from all assemblages [6]. Based on the grouping with the reference sequences, the assemblage was determined.

### Nucleotide sequence accession numbers

*Giardia* sequences generated in this study were deposited in the NCBI GenBank database under the following accession numbers: MW138896-MW138913 (*gdh*), MW138914-MW138934 (*bg*), and MW138935 (*tpi*).

### Statistical analyses

Differences in occurrence of the different assemblages between dog groups were assessed using two-sample tests on the equality of proportions. The cysts per gram (CPG) were determined by qPCR with a calibration curve ranging from 3 × 10^6^ to 300 CPG [25]. The relationship between CPG and assemblages was assessed using negative binomial regression. A cluster-correlated robust variance estimator was included in the analyses to adjust for clustering of dogs at the household, shelter, or hunting dog group level, as described [20]. Statistical analysis was performed using STATA 16 (StataCorp LP, College Station, TX, USA).

## Results

### Diagnostic tests and multilocus genotyping

Of the 189 samples, 107 (56.6%) samples yielded negative MLG or *SSU rDNA* genotyping results. Raw data for *Giardia* results including qPCR Cp, CPG, IDEXX SNAP^®^ *Giardia*, DFA, and CSF test for all samples are provided in Additional file 2: Table S2. CSF results of endoparasites in general are beyond the scope of this article and can be found in Uiterwijk et al. [20]. Table 1 shows detailed results of the 82 samples with *gdh*, *bg*, *tpi*, and/or *SSU rDNA* positive PCR results. Mixed C/D samples are defined as samples that showed double peaks in the sequences. This was shown with the loci *SSU rDNA* (*n* = 8) and *bg* (*n* = 1). Combined mixed C/D results are defined as samples containing assemblage C based on one locus and assemblage D based on another locus, or mixed C/D. Of the 14 combined mixed C/Ds, 8 showed double peaks at the *SSU rDNA* locus at the position where assemblage C and D differ (see Additional file 3: Figure S1), and 6 showed assemblage C with 1 locus and assemblage D with another, including 1 C/D at the *bg* locus (Additional file 2: Table S2). The *SSU*-*rDNA* sequences without a double peak are identical to the GenBank sequences GU126431 (assemblage AI), GU126436 (assemblage C), and GU126442 (assemblage D).

**Table 1:** Distribution of the assemblages AI, C, mixed C/D, and D of MLG (*gdh*, *bg*, and *tpi* loci) and *SSU rDNA*, and combined results

	AI	C	C/D	D	Total
*gdh*	1	6	0	11	18 (9.5%)
*bg*	1	6	1	13	21 (11.1%)
*tpi*	1	0	0	0	1 (0.5%)
*SSU rDNA*	2	17	8	55	82 (43.4%)
Combined	2	17	14	49	82

In three dog groups, assemblage D was most prevalent. In the hunting dogs, both assemblage D and C/D were highly present. Assemblage AI was only present in two household dogs (Table 2).

**Table 2:** Occurrence (%) and 95% CI (in brackets) of *Giardia duodenalis* assemblage AI, C, combined mixed C/D, D, and samples for which no assemblage could be determined, over the dog population

Dog population	AI	C	C/D	D	No assemblages determined
Household *n* = 47	4.3 (1.0-15.8)	6.4 (2.1–17.9)	0	12.8 (5.7–26.0)	76.6 (62.4–86.6)
Clinical *n* = 64	0	15.6 (8.6–26.6)	3.1 (0.8–11.9)	32.8 (22.5–45.1)	48.4 (36.2–60.9)
Sheltered *n* = 48	0	6.3 (1.4–24.0)	0	22.9 (13.5–36.1)	70.8 (57.5–81.4)
Hunting *n* = 30	0	3.3 (1.1–9.9)	40.0 (29.4–51.6)	36.7 (3.3–40.5)	20.0 (16.5–24.0)
Total *n* = 189	1.1 (0.3–4.3)	9.0 (5.4–14.6)	7.4 (2.6–19.4)	25.9 (20.2–32.6)	56.6 (45.6–67.0)
Mean CPG (95% CI)	3.3x10^4^ (1.1x10^3^–6.4x10^4^)	9.3x10^4^ (2.2x10^4^–1.6x10^5^)	6.5x10^4^ (1.0x10^4^–1.2x10^5^)	4.7x10^5^ (4.3x10^4^–9.8x10^5^)	9.1x10^3^ (3.9x10^3^–1.4x10^4^)

Mean CPG and 95% CI (in brackets) are provided. *CI* confidence interval

### Association of assemblage AI with synantropism

When considering only the samples for which an assemblage was determined and adjusting for clustering, the difference in occurrence of assemblage AI between synanthropic dogs (household dogs; 18.2%, 95% CI 4.0–5.4%, *n* = 11) compared to dogs living among other dogs (sheltered and hunting dogs; 0.0%, 95% CI 0.0–10.0%, *n* = 35) was not significant (*P *= 0.131). However, the occurrence of AI was significantly higher among household dogs compared to clinical dogs (0.0%, 95% CI 0.0–9.7%, *n* = 36, two-sample test, *Z *= –2.5813, *P* = 0.010).

### Association of assemblages with loose stool

Considering only the dogs with loose stool, there was no significant difference in the occurrence of AI between the dog groups. Indeed, none of the assemblage groups was significantly associated with loose stool. In the samples in which no assemblage could be determined, the CPG was significantly lower in the samples with loose stool (4.6 × 10^3^, 95% CI 2.7 × 10^3^–6.5 × 10^3^) than in the samples with no loose stool (1.0 × 10^4^, 95% CI 3.6 × 10^3^–1.7 × 104, *Z *= –2.23, *P *= 0.026).

### Association of assemblages with CPG

The CPGs of the samples with assemblages C, combined mixed C/D, or D (Table 2) were significantly higher than the CPG of the samples with undetermined assemblage (*P *= 0.000). CPGs of samples with assemblage D were significantly higher than the samples with assemblage C (*P *= 0.006), AI (*P *= 0.005), and the undetermined (*P *= 0.000) samples, but not with combined mixed C/D.

## Discussion

To determine the (sub)assemblages of *G. duodenalis* shed by dogs and their association with synanthropism, clinical signs, and cyst shedding, we performed MLG and SSU rRNA PCR on qPCR-positive fecal samples from four groups of dogs. In 43.4% of the *G. duodenalis* qPCR-positive samples (*n* = 189), genetic characterization was successful. Due to the high sensitivity of the multicopy *SSU rDNA*, this locus yielded most positive results of the four loci. Canid assemblages C and D were the most prevalent in all dogs groups, which is in accordance with previous findings where dog-specific assemblages were most prevalent in household and sheltered dogs [14, 31–34]. The potential zoonotic assemblage AI is mainly found in pets and livestock, and to a lesser extent in humans [2]. Our findings are therefore also in accordance to reports in which AI was found in dogs [35], even though this assemblage was not most prevalent in our study. Assemblage AI was only detected in the synanthropic household dogs. Because spatiotemporal epidemiological and molecular information on humans and animals needs to be combined to demonstrate zoonotic transmission in a household [36, 37], the sources of AI in those two dogs remain unclear. The dogs may have contracted this genotype directly or indirectly from various sources, e.g. from humans, other dogs, or other animals. In turn, the dogs may infect other animals and people, even though only one dog was shown to have a patent infection (Additional File 2: Table S2). Molecular detection of an assemblage in stool could be due to a patent infection or merely mechanical passage. To detect patent infections, mRNA assays can be used [38] or cysts or cyst wall proteins can be detected in stool samples [25]. In our study, three assays to detect cysts or cyst wall proteins were performed; DFA, CSF, and IDEXX SNAP^®^ *Giardia*. In general, samples which tested positive with DFA, CSF, and/or IDEXX SNAP^®^ *Giardia* showed higher CPG because of the lower sensitivities of these tests compared to qPCR [25]. This means that for samples with relatively lower CPGs, such as from the one AI dog, in many cases it could not be determined whether there was a true infection or merely passage.

The occurrence of assemblage AI was significantly higher among household dogs compared to the (predominantly synanthropic) clinical dogs. Both household dogs that shed AI did not have loose stool. But since none of the assemblage groups was significantly associated with loose stool, this does not indicate that dogs infected with assemblage AI may show less or more symptoms than with assemblages C and/or D. We found significant differences in CPG shed for the different assemblages. Lower sensitivity of MLG and *SSU rDNA* nested PCRs compared to qPCR most likely accounts for the significant difference in CPG between MLG/*SSU rDNA*-negative and -positive samples. Assemblages D and combined mixed C/D showed the highest CPG compared to the other assemblages. This may indicate that infections with AI, C, C/D, and D behave differently in dogs with regard to cyst production. Possibly, canid-assemblages C and especially D are better adapted to dogs, thus leading to higher CPGs and relatively more often positive MLG and *SSU rDNA* results. This might have led to an underreporting of samples with sub-assemblage such as AI. Also, this may contribute to the relatively more reports of canid-assemblages in dogs compared to non-canid assemblages mentioned above [6, 14, 31–35]. Use of Next Generation Amplicon Sequencing might resolve the problem of underreporting of certain assemblages, because with this sequencing method, assemblages present in mixed infection can be separately detected. Also, infections with lower CPG can be detected [38].

The finding of lower CPG in samples with loose stool for which no assemblage could be determined may be explained by the fact that nested PCRs showed higher sensitivity on purified cysts [25] and possibly also on diluted feces (e.g. loose stool). In our study, no assemblage B or other subtypes of assemblage A such as AII were detected, which have been found in dogs before [12, 39, 40]. Traub et al. (2009) defined three transmission cycles of *Giardia* assemblages in dogs: anthroponotic, zoonotic, and dog-specific [41]. Which assemblage prevails in dogs seems to depend on factors related to dogs, such as a high level of coprophagic behavior [42], the level of contact with humans and living conditions, factors related to the parasite, such as host-specific adaptations of assemblages, and factors related to the human population, such as *Giardia* prevalence and circulating assemblages [43]. Once an assemblage is introduced, it may circulate relatively easily among (groups of) dogs. For example, the high prevalence of D and C/D in the hunting dogs in our study can be explained by living conditions and behavior favorable for feco-oral transmission, introduction of dog-specific assemblages due to more contact with canids than with humans, and the relatively low prevalence of *Giardia* in the Dutch human population [44, 45]. Also, this may explain, at least partly, the reports of high *versus* low risks of zoonotic transmission [7, 39, 46, 47].

## Conclusions

*Giardia* infections in both synanthropic and non-synanthropic dogs were mainly caused by canid assemblages, with the potentially zoonotic assemblage AI in just two synanthropic dogs (1.1% of all dogs). Dogs with canid assemblages, especially assemblage D, showed much higher CPGs. This may suggest that the assemblage AI, although capable of infecting dogs, has a different host-pathogen interaction and is possibly less able to multiply in dogs compared to the canid assemblages. Based upon our results, the zoonotic risk of *Giardia* infections in dogs is low.

## Supplementary information

**Additional file 1: Table S1**. Characteristics of multi-locus genotype nested PCRs.

**Additional file 2: Table S2.** Raw data.

**Additional file 3: Figure S1**. Example of SSU-rDNA fragment with “double peaks” in the Sanger sequence results.

## Data Availability

The datasets used and/or analysed during the present study are presented in the article and its additional files, or are available from the corresponding author upon reasonable request.

## Abbreviations

*bg*: Beta-giardin
CI: Confidence interval
CPG: Cyst per gram feces
*gdh*: Glutamate dehydrogenase
MLG: Multi-locus genotyping
*P*: *P*-value
qPCR: Quantitative real-time PCR
*tpi*: Triose phosphate isomerase
*SSU rDNA*: Small subunit ribosomal DNA
VMDC: Veterinary Microbiological Diagnostic Center
