# Supplementary material for: Giardia duodenalis multi-locus genotypes in dogs with different levels of synanthropism and clinical signs
Source: Parasit Vectors. 2020 Dec 2;13:605. doi: 10.1186/s13071-020-04496-2 (PMC7709413; doi:10.1186/s13071-020-04496-2)
Supplement: Supplementary file 1 — Additional file 1: Table S1. Characteristics of multi-locus genotype nested PCRs. [file 13071_2020_4496_MOESM1_ESM.docx]

| Table S1. Characteristics multi-locus genotype nested PCRs | | |  |  |
| --- | --- | --- | --- | --- |
|  |  |  |  |  |
| locus | primers | | length amplicon* | reference |
| *SSU rDNA* | 1st Forward | 5'-TGGAGGAAGGAGAAGTCGTAAC-3' | 315 | 1 |
|  | 1st Reverse | 5'-GGGCGTACTGATATGCTTAAGT-3' |  |  |
|  | 2nd Forward | 5'-AAGGTATCCGTAGGTGAACCTG-3' |  |  |
|  | 2nd Reverse | 5'-ATATGCTTAAGTTCCGCCCGTC-3' |  |  |
|  |  |  |  |  |
| *glutamate dehydrogenase* | 1st Forward | 5'-TTC CGT RTY CAG TAC AAC TC-3' | 530 | 2 |
| (*gdh*) | 1st Reverse | 5'-ACC TCG TTC TGR GTG GCG CA-3' |  |  |
|  | 2nd Forward | 5'-ATG ACY GAG CTY CAG AGG CAC GT-3' |  |  |
|  | 2nd Reverse | 5'-GTG GCG CAR GGC ATG ATG CA-3' |  |  |
|  |  |  |  |  |
| *β-giardin* | 1st Forward | 5'-AAG CCC GAC GAC CTC ACC CGC AGT GC-3' | 511 | 3 |
| (*bg*) | 1st Reverse | 5'-GAG GCC GCC CTG GAT CTT CGA GAC GAC-3' |  |  |
|  | 2nd Forward | 5'-GAA CGA GAT CGA GGT CCG-3' |  |  |
|  | 2nd Reverse | 5'-CTC GAC GAG CTT CGT GTT-3' |  |  |
|  |  |  |  |  |
| *triosephosphate isomerase* | 1st Forward | AAATIATGCCTGCTCGTCG |  | 4 |
| (*tpi*) | 1st Reverse | CAAACCTTITCCGCAAACC |  |  |
|  |  |  |  |  |
| assemblage A specific | 2nd Forward | CGC CGT ACA CCT GTC A | 332 | 5 |
|  | 2nd Reverse | AGC AAT GAC AAC CTC CTT CC |  |  |
|  |  |  |  |  |
| assemblage B specific | 2nd Forward | GTT GTT GTT GCT CCC TCC TTT | 390 | 6 |
|  | 2nd Reverse | CCG GCT CAT AGG CAA TTA CA |  |  |
|  |  |  |  |  |
| * in basepares after 2nd amplification | |  |  |  |

list references Tabel S1

1 Caccio et al., 2010. Identification of Giardia species and Giardia duodenalis assemblages by sequence analysis of the 5.8S rDNA gene and internal

transcribed spacers. Parasitology 137: 919-925

2 Caccio et al., 2008. Multilocus genotyping of Giardia duodenalis reveals striking differences between assemblages A and B. International Journal for Parasitology 38 (13): 1523-1531

3 Tseng et al., 2014. Prevalence and genotype of Giardia duodenalis from faecal samples of stray dogs in Hualien city of eastern Taiwan. Tropical Biomedicine 31(2): 305-311

4 Sulaiman et al., 2003. Triosephosphate isomerase gene characterization and potential zoonotic transmission of Giardia duodenalis. Emerging Infectious Diseases 9 (11): 1444-1452

5 Geurden et al., 2008. Mixed Giardia duodenalis assemblage A and E infections in calves. International Journal for Parasitology 38(2): 259-264.

6 Levecke et al., 2009. Molecular characterisation of Giardiaduodenalis in captive non-human primates reveals mixed assemblage A and B infections and novel polymorphisms. International Journal for Parasitology 39 (14): 1595-1601
