# Supplementary material for: Giardia duodenalis multi-locus genotypes in dogs with different levels of synanthropism and clinical signs
Source: Parasit Vectors. 2020 Dec 2;13:605. doi: 10.1186/s13071-020-04496-2 (PMC7709413; doi:10.1186/s13071-020-04496-2)
Supplement: Supplementary file 3 — Additional file 3: Figure S1. Example of SSU-rDNA fragment with “double peaks” in the Sanger sequence results. [file 13071_2020_4496_MOESM3_ESM.pptx]

## Slide 1
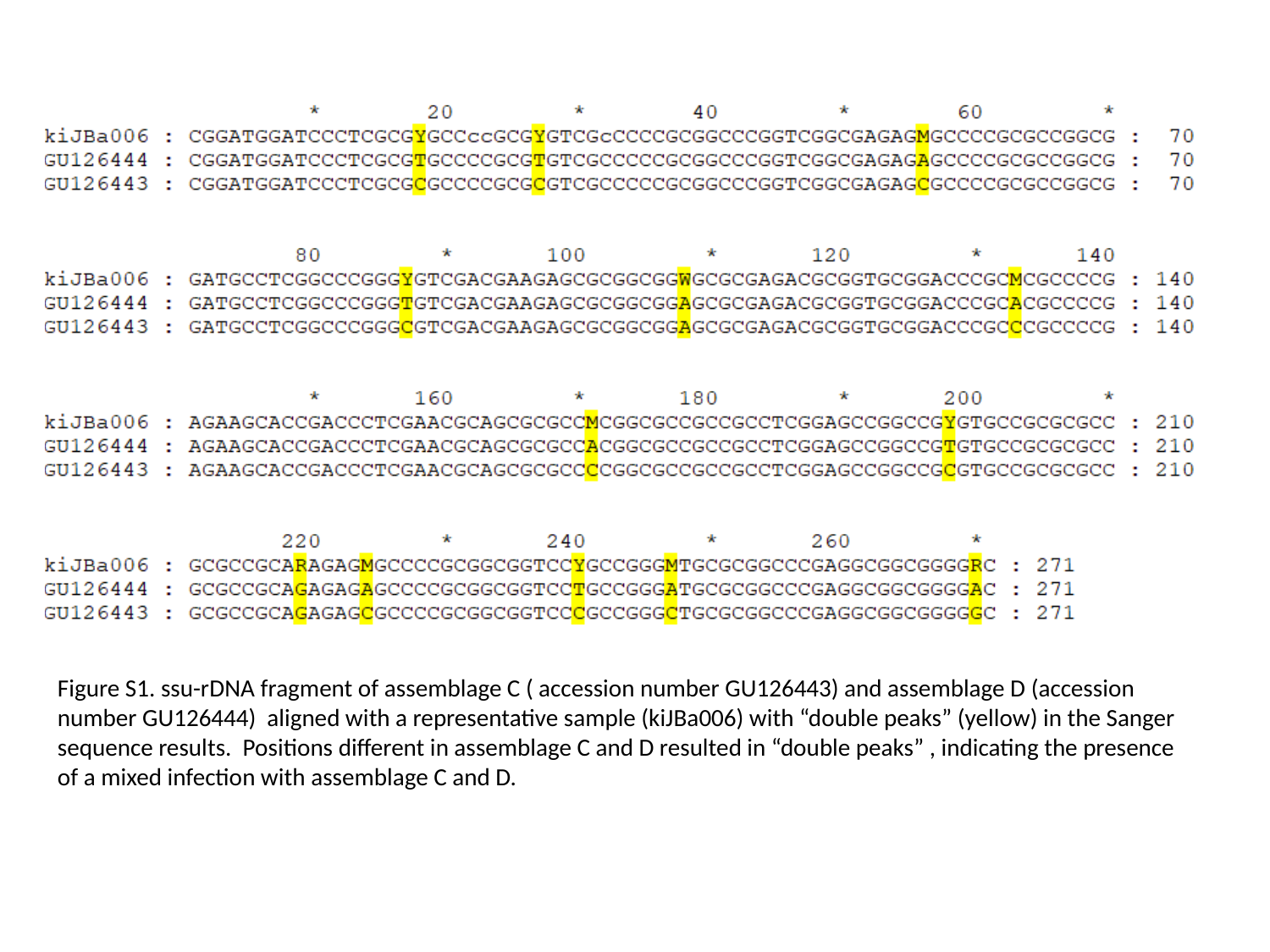

Figure S1. ssu-rDNA fragment of assemblage C ( accession number GU126443) and assemblage D (accession number GU126444) aligned with a representative sample (kiJBa006) with “double peaks” (yellow) in the Sanger sequence results. Positions different in assemblage C and D resulted in “double peaks” , indicating the presence of a mixed infection with assemblage C and D.
